# Notes of an European Tour

**Published:** 1846-03-01

**Authors:** F. H. Hamilton

**Affiliations:** Professor of Surgery in Geneva Medical College


### Notes of an European Tour.---No. 10.

Communicated for the Buffalo Medical Journal, by F. H. HAMILTON, M. D., Professor of Surgery in Geneva Medical College.

PALERMO, SICILY, MAY 30.

From Mr. Lester and his accomplished Lady, I parted with sincere regret. Five or six Americans were at Genoa, and with myself they delayed leaving from day to day, because Mr. Lester had always for them some new engagement of interest or profit; and if his time is forever occupied by idle tourists, he must charge it all to himself. He has made his house an "oasis," at least so it was to me, and he cannot but expect that every pilgrim will leave it with reluctance.

Waiting for a favorable wind we did not go on board our good ship (Lincoln, Capt. Ellis, of Boston,) until 2 P. M. As "compagnons de voyage" we had Mr. and Mrs. L., of New-York, and Mr. S., of Northampton: and with none but Yankees about us,and with the stars and stripes floating above us, we all, in full Congress assembled, declared ourselves "free and independent:" after which we sat down to one of the best dinners of codfish and potatoes, with prime Ohio pork, which ever a hungry man eat.

At four o'clock we were under full canvass, and as we were just leaving the outer mole, Mr. L., for some years a resident of Genoa, called my attention to a small church, situated almost under the southeastern wall of the city, where daily the "illegitimates" meet for prayer and worship. Near by is an embankment, quite upon the shore of the bay, "where" says Mr. L., "are three pits, which descend and communicate with the sea below; each covered with a trap door. Into these are thrown in open day those who die in the Hospitals; being brought thither in a rough box, without lid, with merely a black cloth laid over them, and this often sufficiently displaced as they pass along the public streets, to expose an arm or a knee of the *pauper's* corpse! and when they reach this spot they remove the scanty covering, and thrust his poor bones headlong adown the vault."

May 23d.—All day we have lain becalmed in sight of Elba; indeed we are scarcely a league from shore, which at this distance looks bold, rocky and sterile.

May 24th.—Still we are off Elba, and if the winds of this sea were not proverbially fickle, we should haul down the long boat and go ashore. Most of the day I have spent in dissecting "Portugese Men of War," the "Holuthuria physalis" of Naturalists. We had caught one while crossing the Atlantic, but *these* were to me peculiar in being united by a gelatinous cord side and side with each other, forming thus a long "fleet," varying from one to fifteen yards in length—and which might be termed a "Spanish Armada," rather than a "Portugese Man of War." The Captain has proved himself remarkably expert in capturing these little cruisers, with a pail and rope, and I have had about me most of the day a very attentive class of anatomical students. And indeed we have all been surprised at the beautiful simplicity of this lowest order of animal creation. It is a gelatinous sack, diaphanous, about 2 inches in its longest diameter, ovoid,—the cavity of the sack being filled only with air. At one extremity is a small brown spot, quite hard, and often containing a small insect; this is the stomach. Along the upper part of the "animal" is a fimbriated membrane, which being raised serves as a sail, the color of which when in the water is of a fine pink, or rather iridescent. Again along the lower surface are numerous long, slender filaments, of a light blue and red color, and which are said to sting severely. Whenever the animal was touched it contracted its walls violently, and this it was able to do even after its walls were entirely slit asunder.

25th.—We are again under sail, but beating against a sirocco—now ploughing above the madrepores, along the shores of Corsica—(the coral fishery was once here a considerable trade) now coasting the isles of Pianosa and Monte Christo.

26th.—The blue hills of Sardinia have scarcely been out of sight during the day. The wind is a stout nor-western gale, and although it comes direct from the shores of Sardinia, whose malaria were once deemed so fatal, we give it a hearty welcome, since it tightens the canvass and steady's the keel direct for Palermo.

27th.—Last night we were all summoned on deck to see the fires of Stromboli, nearly 70 miles distant, and which could be distinctly seen giving a short and fitful illumination to the south eastern horizon. Two thousand years or more this mountain has daily and nightly trimmed its lamps and lifted its lights, to warn the mariner of his approach to those islands within whose hollow sides Aeolus locks up the winds. The forge of old "Vulcan," although no farther off than Stromboli, could not be seen; probably it was concealed by the intervening islands.

28th.—After a voyage of five days I am at length snugly quartered in the second story of the "London Hotel"—with cold glazed, earthen floors and a balcony overhanging the piazza.

Our reception at the custom house was on this wise: Being placed upon a short quay between the building and the sea, we were compelled to wait about half an hour, until the American Consul, Mr. Masten, had arrived. A dark mustachoed official then reached a long pair of tongs at full arms length across the iron paling for the Captain's papers, which were immediately thrown into a large sheet iron chest filled with the smoke of burning sulphur. Fifteen minutes were deemed sufficient to kill the "fomites," those little Levantines who are accused of smuggling in the Plague; at least they were so far disabled that the officer could now, taking the papers again in his tongs, approach near enough to read the signatures and the seals without danger. The result was satisfactory and we were now allowed upon the presentation of our several passports, and a slight inspection of luggage, to pass.

I have just returned from Mr. Masten's, where I have spent the evening very pleasantly with a small company of his friends—among whom I was made acquainted with his family physician, Dr. Salemi, one of the

Surgeons to the "Spedale Grande," who is here quite distinguished.—I had brought letters from my friend Dr. Smith, of the Boston Medical Journal, to Dr. Portal, but on enquiring after him I was told by Dr. Salemi that during the last year he had procured the assassination of a rival Surgeon in the Hospital, and that the matter having become public, he was so much "mortified" that he fled to Girgenti, on the opposite side of the island, where some say he killed himself, some believe he was assassinated, others believe he is only secreted. Probably he was assassinated, as these things are only matters of course here—his widow also is in town and wears mourning. I was warned by Mr. Masten, never, while in Sicily, to go out alone after dark.

29th.—Have been with Dr. Salemi to the "Grand Hospital," containing about 400 patients. Dr. Salemi here made me acquainted with Dr. Gogolguna, the chief Surgeon, and three Physicians—they are all fine, intelligent looking men, and were exceedingly polite in showing me every thing of interest in their several wards. The circular amputation is generally performed, and the French practice in the closure of large wounds, is usually adopted. Many cases of chronic, indolent ulcers—saw none treated by straps—generally some ointment, over which a dirty roller, &c. Two cases of "Lepra" proper—not here considered contagious, nor incurable, but yielding generally to medicated baths, regimen &c.; looks in the early stage much like "scabies." Several cases of "Lepra tuberculosa," covering and painfully disfiguring the whole features, were shown me; also a few cases of "Pellagra," which is however by no means as common here as in Lombardy. Syphilis abounds, and is treated both with and without mercurials, the plan being eclectic. Intermittents are common in the autumn and winter months.

Dr. Gogolguna informed me that the climate of Palermo and its vicinity was generally considered remarkably healthy, and that the temperature was even and agreeable, being protected in a great measure from the sirocco by a high ampitheatre of hills on the south, and the sea softening the winds which blow from the north. The valley in which Palermo stands, is the "Aurea Valle" and "Hortus Siciliæ of the ancients—producing nearly all the plants which can be found in the Tropics. Within three miles of the walls of the city, I have seen orange, lemon and olive groves—the palm, fig and date tree—the centaury plant growing wild—the black pepper tree—while the surface of the earth blooms with red poppies, marigolds and a thousand other gaudy flowers.

But while speaking of the beauty, fertility and general salubrity of this spot, (being especially fitted for a tuberculous patient,) I must not forget the fearful visitations of the plague to which it has been from time to time subjected, nor the later scourge of the Eastern Cholera in 1837, to which Mr. Gardner, the then American Consul, was one of the first victims,—when from a population of 200,000, 40,000, or one-fifth of the whole were swept off; until there was neither room nor time to bury the dead, and they were carried out and piled in large heaps at the foot of Monte Pelligrino. Placed here, as I suppose, to excite the sympathies of the patron Saint of Palermo, "Santa Rosalia," whose gilded grotto is at the summit of the mountain, and in honor of whom once a year the citizens celebrate a great festival. Santa Rosalia was the young and beautiful niece of "William the Good," when in 1159 she retired to this grotto to end her life as a Monastic. And more than once has this fair Saint, when her bones have been carried three times in solemn procession about the city, rescued its inhabitants from the ravages of the plague.

On leaving the Hospital my attention was called to a fresco painting on the walls of the principal Court, underneath an arcade, representing "Death on the pale Horse." It is now much broken and defaced, but the white horse, mounted by a skeleton, is easily traced. Underneath is the following inscription: "O mors quam amara est tua." It was painted by Antonio Crescenzio, and is evidently many centuries old; and as West is known to have visited Palermo before he executed his great work bearing the same name, it is not improbable that he took the hint from this painting, yet in my opinion he has infinitely improved upon the old master.

30th.—Returning to-day from Monreale, I stopped at the Convent of the Capuchins, about a mile outside the southwestern gate. The Capuchins are a class of Monks who live by begging solely, and are seen every where in the streets of Palermo, with bare feet, bare and bald heads, long beards, coarse brown cloaks secured to the waist by a rope, and with a greasy, filthy looking bag, containing fragments of food. They live however in a large and princely building.

Francesco, my guide, having made application to visit the Catacombs at the principal door of the Convent, we were led by one of the brethren carrying a lighted taper, through the chapel and down a pair of stone steps into a suit of long stone corridors, or subterranean galleries; of which I believe there are five, containing altogether, I was told, about 6,000 mummies. All the members of the order are entitled to a burial here, and also such other good Catholics as pay for the privilege. For a shelf or a niche the charge is about four dollars, and for drying about one dollar. The bodies are dried by laying them in a small stone vault, of which there are many along the galleries, and keeping them closely locked during six months. They are then removed, and their friends, if they have any, clothe them according to their fancy, and deposit them, if females, upon a shelf with the face toward the spectator, or if they are males, they are placed in the upright posture against the wall, and properly secured in place. Thus as you pass through the long dark vaults, you walk between rows of cowled capuchins and more gaily mantled citizens, with glaring sockets, and fallen jaws, and black and mouldering faces, looking like the royal guard of his Majesty, the King of the Infernal Regions. The dress of the females is often exceedingly fantastic; one is clad in a rich silk gown with an embroidered belt of a different color, secured with a gold buckle—on her wrists are handsome bracelets, and upon her feet a pair of pink satin slippers, while her mummified face is tastefully set off with a lace cap and pink rosettes.—Francisco has lately deposited one of his own children here, a daughter, aged 7 years; and with much emotion he conducted me to the disgusting object, which, he said, was once a beautiful child—he adjusted the little cap and replaced affectionately a stray lock of the black hair, crossing himself meanwhile and ejaculating a short prayer for her deliverance from purgatory.

The Capuchin who accompanied us was exceedingly attentive and amusing, bursting now and then into a broad laugh, as he showed us some of the upright bodies whose heads had fallen off—or those whose lower jaws were much dropped, in one of which was a tongue, curled up and dried like ham. The label attached—all are labelled, the label announcing the name, profession, rank and time of death—informed us that he died in the year 1693, according to which he should have been removed from his present position to the general receptacle under the pavements, last year ; since it is a rule that the bodies shall be thus exposed for the benefit of their friends, one hundred and fifty years, and after that they shall be buried. Another body was shown us which during life had held communication with Angels !—But the most wonderful thing of all, and which has become of late a source of no small revenue to the treasury of these shrewd beggars, is yet to be seen. Behind a wire screen, in a niche, is placed the body of Signor and Conte D'Americo Amari, who died in 1766. On his right (without the screen) is the body of his son, and on his left the body of his grandson, the latter having died in 1838, and now there is a miracle ! "For all" says the Capuchin "who have fevers, and come here, and touch three times either of these bodies are certainly cured !" "Is it so?" said I. "How do you know that?"—"Because" replied the Capuchin, "I see them cured myself almost every day." "How many have you seen cured?" I enquired. "At least five hundred," said he gravely.

When we were leaving the gate of the Convent we met without, several miserable, sickly looking lazzarones, who were waiting, they said, "to be cured of their fevers."

